# Prevalence and factors associated with multidrug-resistant tuberculosis in South India

**DOI:** 10.1038/s41598-020-74432-y

**Published:** 2020-10-16

**Authors:** Smita S. Shivekar, Venkatesh Kaliaperumal, Usharani Brammacharry, Anbazhagi Sakkaravarthy, C. K. Vidya Raj, Chitra Alagappan, Muthuraj Muthaiah

**Affiliations:** 1 Department of Microbiology, State TB Training and Demonstration Centre, Government Hospital for Chest Diseases, Puducherry, India; 2 Department of Microbiology, Jawaharlal Institute of Postgraduate Medical Education and Research, Puducherry, India; 3 Department of Genetics, Dr.A.L.M. Postgraduate Institute of Basic Medical Sciences, University of Madras, Tamil Nadu, Chennai, India; 4 Department of Environmental Science, Central University, Kasargod, Kerala India

**Keywords:** Genetics, Microbiology, Molecular biology, Diseases

## Abstract

India accounts for about one-fourth of the global burden of MDR-TB. This study aims to assess  the prevalence and factors associated with tuberculosis drug resistance among patients from South India. MTBDRplus assay and MGIT liquid culture performed on 20,245 sputum specimens obtained from presumptive MDR-TB cases during a six-year period from 2013 to 2018 were analyzed retrospectively. Univariate and multivariate logistic regression analysis was carried out to evaluate factors associated with MDR, Rifampicin mono-resistance, and Isoniazid mono-resistance. MDR, Rifampicin mono- resistant and Isoniazid mono-resistant TB were  found in 5.4%, 2.5%, and 11.4% cases of presumptive MDR-TB, respectively. Based on *the rpoB* gene, true resistance, hetero-resistance, and inferred resistance to Rifampicin was found in 38%, 29.3%, and 32.7% of the 1582 MDR cases, respectively. S450L (MUT3) was the most common *rpoB* mutation present in 59.4% of the Rifampicin resistant cases. Of the 3390 Isoniazid resistant cases, 72.5% had mutations in the *katG* gene, and 27.5% had mutations in the *inhA* gene. True resistance, heteroresistance, and inferred resistance accounted for 42.9%, 22.2%, and 17.3% of the 2459 *katG *resistant cases, respectively. True resistance, heteroresistance, and inferred resistance for the *inhA* gene were found in 54.5%, 40.7%, and 4.7% cases, respectively. MDR-contact (AOR 3.171 95% CI: 1.747–5.754, p-0.000) treatment failure (AOR 2.17595% CI: 1.703–2.777, p-0.000) and female gender (AOR 1.315 95% CI: 1.117–1.548, p-0.001), were positively associated with MDR-TB. Previous TB treatment did not show a significant positive association with MDR (AOR 1.113 95% CI: 0.801–1.546, p-0.523). Old age (AOR 0.994 95% CI: 0.990–0.999, p-0.023) and HIV seropositivity (AOR 0.580 95% CI: 0.369–0.911, p-0.018) were negatively associated with MDR-TB. Although Rifampicin mono-resistance had a positive association with treatment failure (AOR 2.509 95% CI: 1.804–3.490, p < .001), it did not show any association with previous TB treatment (AOR 1.286 95% CI: 0.765–2.164, p-0.342) or with history of contact with MDR-TB (AOR 1.813 95% CI: 0.591–5.560, p-0.298). However, INH mono-resistance showed a small positive association with the previous history of treatment for TB (AOR 1.303 95% CI: 1.021–1.662, p-0.033). It was also positively associated (AOR 2.094 95% CI: 1.236–3.548, p-0.006) with MDR-TB contacts. Thus INH resistance may develop during treatment if compliance has not adhered too and may be easily passed on to the contacts while Rifampicin resistance is probably due to factors other than treatment compliance. MDR-TB, i.e. resistance to both Rifampicin and Isoniazid, is strongly correlated with treatment failure, spread through contact, and not to treatment compliance. The temporal trend in this region shows a decrease in MDR prevalence from 8.4% in 2015 to 1.3% in 2018. A similar trend is observed for Rifampicin mono-resistance and Isoniazid mono-resistance, pointing to the effectiveness of the TB control program. The higher proportion of inferred resistance observed for Rifampicin compared with INH may indicate a surfeit of mechanisms that enable rifampicin resistance. Association of MDR-TB with age, gender, and HIV status suggest the role of the immune system in the emergence of the MDR phenotype.

## Introduction

Tuberculosis is the foremost cause of death from single infectious agent *Mycobacterium tuberculosis*. About 10 million people worldwide were infected in 2018^1^. According to the World Health Organization, 27% of the global TB cases are from India. Besides, India also accounts for 27% of the worldwide burden of rifampicin-resistant TB^2^. The incidence of TB is highest in the 15–24 year age group in India. The incidence rates in men, women, and children were 60%, 34%, and 6%, respectively^2^. A decreasing trend is observed in the TB incidence and mortality in India and other South-East Asian countries like Vietnam and Myanmar. But, Multi-drug resistant tuberculosis (MDR-TB) with resistance to first-line anti-TB drugs viz. Rifampicin and Isoniazid drugs pose a serious threat to the End TB initiative^3^. The global incidence of MDR-TB is 3.4% in new cases and 18% in previously treated cases. Globally, 78% of the rifampicin-resistant TB (RR-TB) cases were multidrug-resistant. Indian government survey from 2014 to 2016 estimated the incidence of MDR-TB as 2.84% in new cases and 11.6% among previously treated patients^4^. Further, rifampicin mono-resistance was negligible, and INH resistance was invariably associated with rifampicin resistance. Worldwide, INH mono-resistance in new cases is 7.2% and 11.6% in previously treated TB cases. In India, INH mono-resistance was observed in 3.8% and 7.8% of new and previously treated cases, respectively.

Many studies on TB drug resistance are conducted using small sample size, and hence the results may not be extended to a larger population. This study analyzed more than twenty thousand sputum positive and positive culture specimens and described the temporal profile of TB drug resistance from 2013 to 2018 in the state of Tamil Nadu and Puducherry, located in the southern part of India. The association between age, gender, previous treatment/failure, HIV status, and drug resistance were also examined. Treatment of MDR-TB involves toxic and expensive drugs and has a lower success rate of about 56% only. Therefore, it is imperative to assess the burden and pattern of MDR-TB and other factors associated with drug resistance, to plan intervention, prevention, treatment, and to evaluate the outcome.

## Materials and methods

### Clinical specimens

The Intermediate Reference Laboratory in Government Hospital for Chest Diseases, Puducherry, India routinely receives specimens for diagnostic workup from Tamil Nadu and Puducherry state since 2013. This retrospective cross-sectional study analyzed clinical and laboratory data collected between January 2013 and December 2018 from 20,245 patients diagnosed as presumptive MDR-TB attending primary healthcare clinics. Specimens were received as per the guidelines described in the Revised National TB Control Programme. The smear-positive sputum samples by fluorescence microscopy were directly processed by GenoType MTBDRplus assay version 2.0 (Hain Life-science, Nehren, German). All the smear-negative sputum samples were processed in the BACTEC MGIT 960 system, and the culture-positive tubes were then processed by GenoType MTBDRplus assay version 2.0. The laboratory requisition form had details regarding the patient’s age, gender, address, treatment history, and HIV status. The study protocol was approved by the Ethical Committee for Intermediate Reference Laboratory of Government Hospital for Chest Diseases, and written informed consent was obtained from each study subject. All methods were applied in accordance with relevant guidelines and regulations.

Sputum samples were refrigerated immediately after collection in primary healthcare clinics and transported at 4 °C to the Intermediate Reference Laboratory at Government Hospital for Chest Disease within 24 h. Sputum decontamination using 4% NALC-NaOH was performed to minimize commensal bacterial flora. The decontaminated sputum specimens were was subjected to microscopy using Auramine O Fluorescent staining.

### Genotype MTBDRplus assay

The GenoType MTBDRplus VER 2.0 is a DNA-strip- based in-vitro assay for identifying the *M.tuberculosis* complex and its resistance to rifampicin (RIF) isoniazid (INH) from smear-positive pulmonary sputum samples and positive culture samples. There are three steps, namely DNA extraction, multiplex PCR amplification with biotinylated primers, and reverse hybridization. Each DNA-strip has five control zones, conjugate control to check the binding of conjugates on strips while doing conjugation process, amplification control to monitor the success of amplification process, and three locus control to check the sensitivity of the reaction for each of the tested gene (*rpoB, katG*, and *inhA*) loci.

The sputum smear-positive samples and bacterial grown on MGIT tubes were decontaminated using the NALC/NaOH method^5^ and centrifuged at 4000 rpm for 20 min in a refrigerated centrifuge. After decontamination, the cell pellet was suspended in 1 to 1.5 ml of sterile phosphate buffer solution. 500 µl suspension of the pellet in phosphate buffer was transferred to a sterile pre-labelled 1.5 ml screw cap micro centrifuge tube and centrifuged at 10,000 rpm for 15 min. The supernatant was discarded and the pellet was suspended in 100 µl Lysis buffer (A-LYS) using a vortex mixer. The tube was incubated at 95 °C in a hot air oven for 5 min, and 100 µl of Neutralisation Buffer (A-NB) was added to each tube. After vortex, the sample was centrifuged at 10000 rpm for 5 min. Approximately 40–80 µl of DNA supernatant was transferred to a fresh sterile screw cap 1.5 ml tube and stored for further amplification process. 5–10 µl of DNA supernatant was transferred to pre-labelled sterile PCR tube containing 40–45 µl of amplification mixes (AM-A and AM-B). 5 µl of sterile Milli-Q water and 5 µl of DNA supernatant from H37Rv were transferred to PCR tubes containing 45 µl of amplification mixes for negative and positive control, respectively. The multiplex PCR reaction for *rpoB*, *katG*, and *inhA* gene loci was performed in a 2720 thermal cycler (Applied Biosystems Inc) under the following conditions; initial denaturation at 95 °C for 15 min; 20 cycles of 95 °C for 30 s, 65 °C for 2 min; 30 cycles of 95 °C for 25 s, 50 °C for 40 s, 70 °C for 40 s; and final elongation at 70 °C for 8 min. The amplicons were stored at 4 °C until further use.

20 µl DEN (denaturing solution) was pipetted out into each well of GT Blot tray and 20 µl of the amplicon was added to each well and mixed well and incubated for 5 min at room temperature. 1 ml of HYB (hybridization solution) was added to each well and gently shaken to homogenize the solution. The pre-labelled DNA-strip was placed into each well with coloured marker facing up. The tray was placed on GT-Blot and incubated for 30 min at 45 °C. After incubation, HYP solution was carefully pipetted with with individual sterile pasture pipette into a beaker containing diluted bleach solution.1 ml STR solution (stringent buffer) was added into each well and incubated for 15 min at 45 °C in GT-Blot. After incubation, STR was carefully pipetted out with individual sterile pasture pipette into a beaker containing a diluted bleach solution. 1 ml RIN (Rinse solution) was added to each well and incubated for 1 min at 25 °C on GT-Blot. After completion of incubation period, the solution was carefully pipetted out with individual sterile pasteur pipette. 1 ml diluted conjugate (Con D-1: 100 ratios) was added into each well and incubated for 30 min at 25 °C on GT-Blot. The solution was aspirated with individual sterile pasture pipette and washed for 1 min at 25 °C with 1 ml RIN per well on GT-Blot. The RIN solution was removed with individual sterile pasture pipette and washed with 1 ml sterile distilled Milli-Q water. 1 ml diluted substrate (Sub D-1: 100 ratios) was added into each well after complete removal of water and incubated for 3 min at 25 °C on GT-Blot. The reaction was stopped as soon as bands are visible by briefly rinsing twice with distilled water. The DNA-strips were removed from the tray using tweezers and dried between two layers of absorbent paper^6^.

### MGIT culture and identification

The MGIT PANTA was reconstituted with 15.0 ml of MGIT growth supplement and mixed well to dissolved completely. 0.8 ml of this enrichment was added to each pre-labelled (specimen) MGIT medium tube and Quality control tube before the specimen's inoculation. All sputum specimens were digested and decontaminated by the standard N-acetyl-l-cysteine-NaOH method. The deposits were suspended in 1 ml sterile phosphate-buffered saline (pH 6.8), and 0.5 ml of the processed specimen was inoculated into MGIT 960 tube and supplemented as recommended by the manufacturer^7^. 0.5 ml of diluted (1:100 ratio) reference strains suspension into quality control tubes. Immediately recapped the tube tightly and mixed carefully by inverting the tube several times. All inoculated MGIT tubes were incubated within the MGIT 960 instrument either until they were flagged positive or for a maximum of 6 weeks. All positive MGIT vials were confirmed for acid-fast bacilli by Ziehl–Neelsen staining and subjected to identification of *M. tuberculosis* complex using rapid immuno-chromatographic test^8^.

## Results

**Figure 1:**
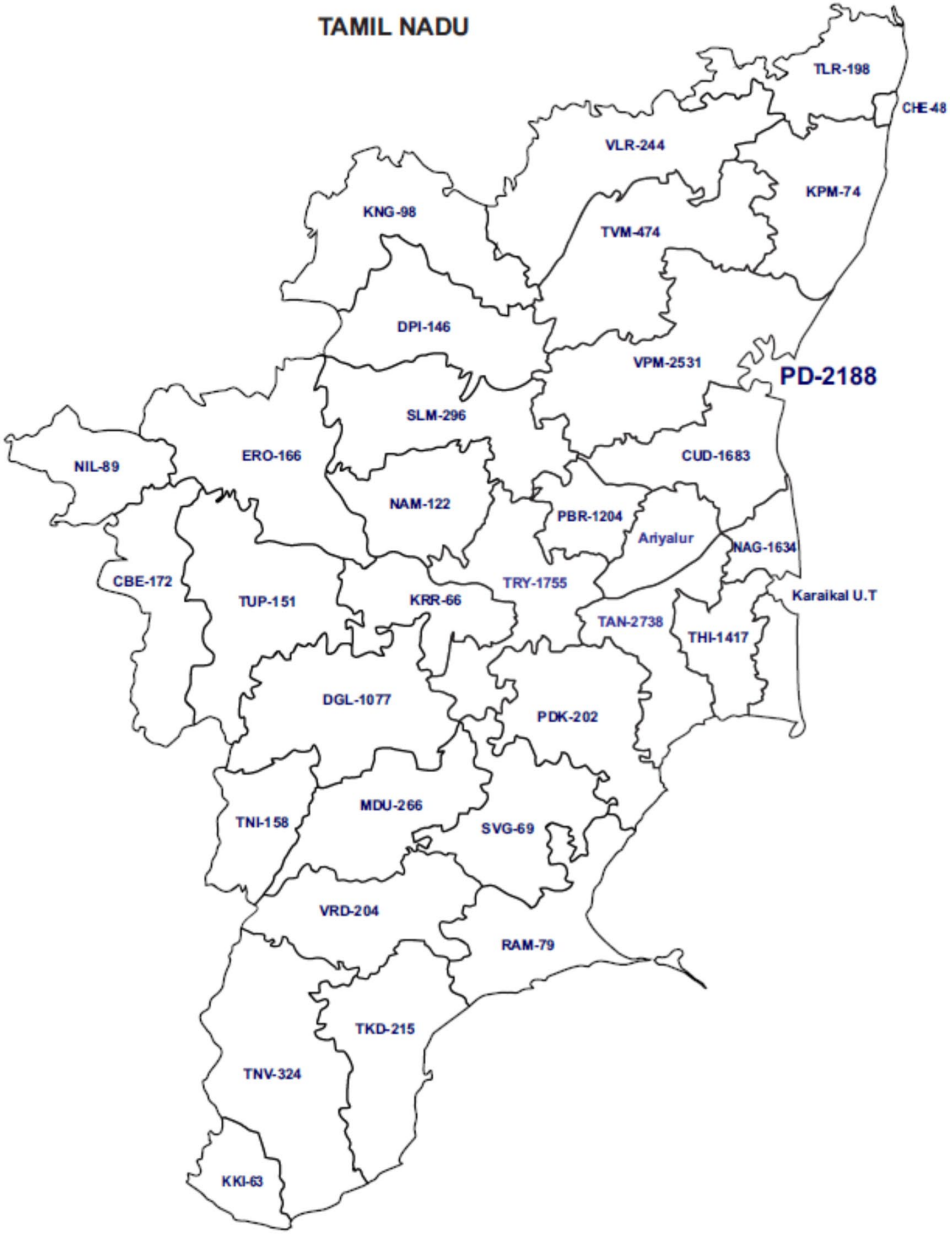
Geographic distribution of samples across different districts of Tamil Nadu and Puducherry (PD) states. (Courtesy: www.d-maps.com, webmaster@d-maps.com).

**Figure 2:**
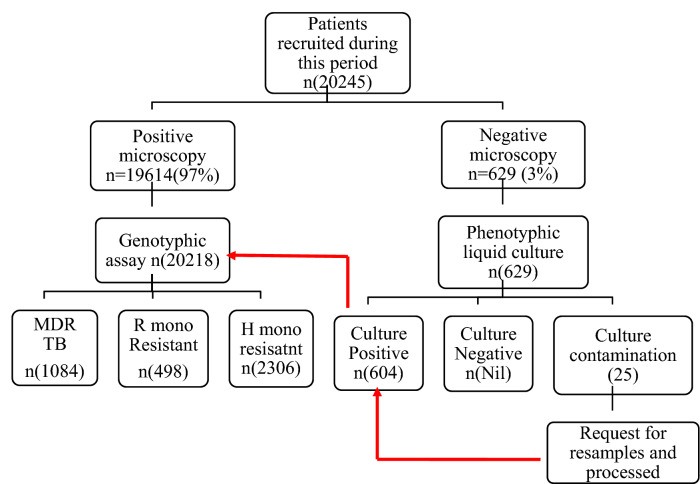
Flow Chart describing the workflow for the study.

**Figure 3:**
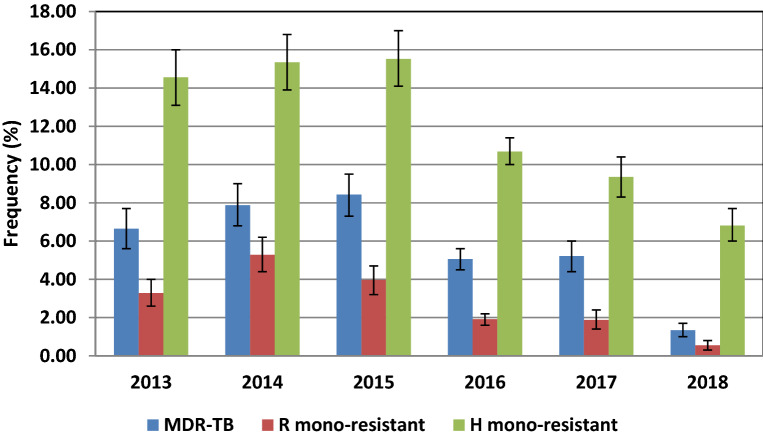
Temporal profile of TB drug resistance in presumptive MDR-TB patients during 2013 to 2018.

Twenty thousand two hundred forty-five specimens were included in this study from the Southern Indian states of Tamil Nadu and Puducherry (Fig. 1). The majority of the specimens analyzed were from Puducherry and its neighbouring districts. All positive sputum specimens were subjected to direct genotyping assay. The negative sputum specimens were cultured, and all the 629 culture-positive isolates were genotyped (Fig. 2). The temporal profile of tuberculosis drug resistance from 2013 to 2018 is depicted in Fig. 3. From the year 2015 onwards, a decreasing trend is observed in the prevalence of tuberculosis drug resistance. Frequency distribution of the prevalence and pattern of molecular drug resistance based on *rpoB*, *katG*, and *inhA* genes is shown in Table 1. The *rpoB* and *katG* and *inhA* gene mutations were observed in 7.8% (95% CI: 7.4–8.2), 12.1% (95% CI: 11.7–12.6) and 4.6% (95% CI: 4.3–4.9) of the cases respectively. Of the 1582 rifampicin resistance cases, 518 (32.7%) were subcategorized as inferred resistance (absence of WT probes without the expression of the mutant probe), 463(29.3%) as hetero-resistance (presence of WT probe with the expression of the mutant probe), and 601 as true resistance (absence of WT probe with the expression of corresponding mutant probe). The codon S450L (MUT3) in the rpoB gene is the most common mutation associated with rifampicin resistance. Of the 3390 isoniazid drug resistance cases, 2459 (72.5%) had mutations in the *katG *gene, and 27.5% (931/3390) had mutations in the *inhA* gene. Of the 2459 *katG* mutants, 30.5%, 59.2%, and 32.7% were subcategorized as hetero-resistance, true resistance, and inferred resistance, respectively. The *katG* S351T mutation is the most common mutation associated with isoniazid drug resistance. Of the 931 *inhA* gene mutants, 40.7% and 54.6% were hetero-resistant and true resistant, respectively. Resistance was inferred in only 4.7% of cases. The promoter region C15T mutation is found in a majority of the *inhA* resistant cases. Isoniazid inferred resistance for (8.7%) was lower than the inferred resistance (32.7%) for rifampicin.

**Table 1:** Prevalence and pattern of molecular drug-resistance based on MTBDRplus assay.

Gene/Type of resistance	Frequency (n)	95% CI
***rpoBgene*** (total)	1582	**7.4–8.2%**
Inferred resistant	518(32.7%)	2.3–2.8%
Hetero-resistant	463(29.3%)	2.1–2.5%
D435V	39(8.4%)	
H445Y	59(12.7%)	
H445D	45(9.7%)	
S450L	320(69.1%)	
True resistant	601(38.0%)	2.7–3.2%
D435V	31(5.2%)	
H445Y	42(7.0%)	
H445D	29(4.8%)	
S450L	499(83.0%)	
***katGgene*** (total)	2459	**11.7–12.6%**
Inferred resistant	253(7.5%)	1.1–1.4%
Hetero-resistant	751(22.2%)	3.4–4.0%
True resistant	1455(42.9%)	6.8–7.6%
***inhAgene*** (total)	931	**4.3–4.9%**
Inferred resistant	44(1.3%)	0.2–0.3%
Hetero-resistant	379(11.2%)	1.7–2.1%
C15T	369(97.4%)	
A16G	0	
T8C	4(1.1%)	
T8A	6(1.2%)	
True resistant	508(42.9%)	2.3–2.7%
C15T	490(96.5%)	
A16G	1(0.2%)	
T8C	8(1.6%)	
T8A	9(1.7%)	

The distribution of MDR-TB, Rifampicin mono-resistant TB, and Isoniazid mono-resistant TB with respect to age, gender, HIV status, and previous treatment are tabulated in Table 2. Our study sample involved a high proportion of males belonging to the age group 18–6045 years. The number of female patients included in the study was 3029 (15%). The majority of cases had a history of previous treatment (95.3%) and were HIV negative (97%). A considerable number of cases had a prior history of contact with MDR-TB (n = 124) or were seropositive for HIV (n = 613).

**Table 2:** Frequency distribution of molecular drug resistance in relation to demographic and clinical characteristics.

Variables	Category	Frequency (%)	Multi drug resistant (%)		Rifampicin mono resistant (%)		Isoniazid mono resistant (%)	
			Yes	No	Yes	No	Yes	No
**Gender**								
	Male	17,216(85)	875(5.1)	16,341(94.9)	422(2.5)	16,794(97.5)	1984(11.5)	15,232(88.5)
	Female	3029(15)	209(6.9)	2820(93.1)	76(2.5)	2953(97.5)	322(10.6)	2707(89.4)
**Age group**								
	< 18 years	256(1.2)	14(5.5)	242(94.5)	11(4.3)	245(95.7)	27(10.5)	229(89.5)
	18–45 years	10,633(52.5)	605(5.9)	10,038(94.1)	260(2.4)	10,383(97.6)	1197(11.2)	9446(88.8)
	46–60 years	7313(36.1)	361(4.9)	6952(95.1)	178(2.4)	7135(97.6)	860(11.8)	6453(88.2)
	> 60 years	2033(10)	104(5.1)	1929(94.9.)	49(2.4)	1984(97.6)	222(10.9)	1811(89.1)
**History of TB**								
	Treated	19,300(95.3)	1027(5.3)	18,273(94.7)	479(2.5)	18,821(97.5)	2211(11.5)	17,089(88.5)
	Naïve	945(4.7)	57(6.0)	888(94.0)	19(2.0)	926(98.0)	95(10.1)	850(89.9)
**Suspect’s criteria**								
	Failure	727(3.6)	78(10.7)	649(89.3)	41(5.6)	686(94.4)	95(13.1)	632(86.9)
	R Rx S ( +)ve at 4^th^ month	644(3.2)	31(4.8)	613(95.2)	24(3.7)	620(96.3)	77(12.0)	567(88.0)
	MDR TB Contact	124(0.6)	18(14.5)	106(85.5)	4(3.2)	120(96.8)	21(16.9)	103(83.1)
	S ( +) Re Rx case	11,815(58.4)	585(5.0)	11,230(95.0)	313(2.6)	11,502(97.4)	1282(10.9)	10,533(89.1)
	Any follow up S ( +)ve	4785(23.6)	272(5.7)	4513(94.3)	70(1.5)	4715(98.5)	613(12.8)	4172(87.2)
	S (-) Re Rx case	716(3.5)	41(5.7)	675(94.3)	13(1.8)	703(98.2)	88(12.3)	628(87.7)
	HIV TB cases	613(3.0)	20(3.3)	593(96.7)	18(2.9)	595(97.1)	56(9.1)	557(90.9)
Total		20,245	1084(5.4)	19,161(94.6)	498(2.5)	19,747(97.5)	2306(11.4)	17,939(88.6)

MDR-TB based on line probe assay was detected in 1084 cases (5.35%; 95% CI; 5–5.7).Contact with MDR-TB (AOR 3.171 95% CI: 1.747–5.754, p-0.000), Treatment failure (AOR 2.17595% CI: 1.703–2.777, p-0.000) and female gender (AOR 1.315 95% CI: 1.117–1.548, p-0.001) were positively associated with MDR-TB (Table 3). The previous history of TB treatment (p = 0.523) did not show a statistically significant association with MDR-TB. Old age (AOR 0.994 95% CI: 0.990–0.999, p-0.023) and HIV seropositivity (AOR 0.580 95% CI: 0.369–0.91, p-0.018) had a negative association with MDR-TB. The number of cases showing mono-resistance to Rifampicin was 498 (2.45%; 95% CI: 2.2–2.7%). Treatment failure is positively associated with Rifampicin mono-resistance (AOR 2.509 95% CI: 1.804–3.490,p-0.000) (Table 4). Isoniazid mono-resistance is positively associated with MDR contact (AOR 2.094 95% CI: 1.236–3.548, p-0.006) and previous treatment (AOR 1.303 95% CI: 1.021–1.662, p-0.033) (Table 5).

**Table 3:** Analysis of factors associated with multidrug resistance in presumptive MDR-TB patients (n = 20,245).

Variables	MDR-TB (n = 1084)	non MDR-TB (n = 19,161)	Crude OR (95% CI)	P-value	Adjusted OR (95% CI)	P-value
**Age**			0.992(0.987–0.997)	0.001	0.994(0.990–0.999)	0.023
**Gender**						
Female	209(6.9%)	2820(93.1%)	1.384(1.184–1.168)	0.000	1.315(1.117–1.548)	0.001
Male	875(5.1%)	16,341(94.9%)				
**Previous history of TB treatment**						
Yes	1027(5.3%)	18,273(94.7%)	0.876(0.665–1.153)	0.340	1.113(0.801–1.546)	0.523
No	57(6.0%)	888(94.0%)				
**Known MDR contact**						
Yes	18(14.5%)	106(85.5%)	3.035(1.835–5.022)	0.000	3.171(1.747–5.754)	0.000
No	1066(5.3%)	19,055(94.7%)				
**HIV status**						
Sero-positive	20(3.3%)	593(96.7%)	0.589(0.375–0.923)	0.021	0.580(0.369–0.911)	0.018
Sero-negative	1064(5.4)	18,568(94.6%)				
**Failure**						
Yes	78(10.7)	649(89.3%)	2.212(1.734–2.821)	0.001	2.175(1.703–2.777)	0.000
No	1006(5.2)	18,512(94.8%)

**Table 4:** Analysis of factors associated with rifampicin mono-resistance in presumptive MDR-TB patients (n = 20,245).

Variables	R resistant (n = 498)	R sensitive (n = 19,747)	Crude OR (95% CI)	P-value	Adjusted OR (95% CI)	P-value
**Age**			1.001 (0.995–1.008)	0.681	1.003(0.995–1.011)	0.478
**Gender**						
Male	422(2.5%)	16,794(97.5%)	1.024(0.800–1.312)	0.850	1.041(0.806–1.344)	0.757
Female	76(2.5%)	2953(97.5%)				
**Previous history of TB treatment**						
Yes	479(2.5%)	18,821(97.5%)	1.240(1.324–1.971)	0.362	1.286(0.765–2.164)	0.342
No	19(2.0%)	926(98.0%)				
**Known MDR contact**						
Yes	4(3.2%)	120(96.8%)	1.324(0.487–3.601)	0.582	1.813(0.591–5.560)	0.298
No	494(2.5%)	19,627(97.5%)				
**HIV status**						
Sero-positive	18(2.9%)	595(97.1%)	1.207(0.749–1.946)	0.440	1.277(0.790–2.066)	0.318
Sero-negative	480(2.4%)	19,152(97.6%)				
**Failure**						
Yes	41(5.6%)	686(94.4%)	2.493(1.795–3.462)	0.000	2.509(1.804–3.490)	0.000
No	457(2.3%)	19,061(97.7%)

**Table 5:** Analysis of factors associated with isoniazid mono-resistance in presumptive MDR-TB patients Suspected cases (n = 20,245).

Variables	H resistant (n = 2306)	H sensitive (n = 17,939)	Crude OR (95% CI)	P-value	Adjusted OR (95% CI)	P-value
**Age**			1.002(0.998–1.005)	0.305	1.001(0.998–1.005)	0.491
**Gender**						
Male	1984(11.5%)	15,232(88.5%)	0.913(0.806–1.034)	0.154	0.927(0.815–1.054)	0.245
Female	322(10.6%)	2707(89.4%)				
**Previous history of TB treatment**						
Yes	2211(11.5%)	17,089(88.5%)	1.158(0.932–1.438)	0.185	1.303(1.021–1.662)	0.033
No	95(10.1%)	850(89.9%)				
**Known MDR contact**						
Yes	21(16.9%)	103(83.1%)	1.591(0.993–2.550)	0.053	2.094(1.236–3.548)	0.006
No	2285(11.4%)	17,836(88.6%)				
**HIV status**						
Sero-positive	56(9.1%)	557(90.1%)	0.777(0.588–1.026)	0.075	0.788(0.596–1.042)	0.095
Sero-negative	2250(11.5%)	17,382(88.5%)				
**Failure**						
Yes	95(13.1%)	632(86.9%)	1.177(0.944–1.466)	0.148	1.164(0.933–1.451)	0.178
No	2211(11.3)	17,307(88.7%)				

**Figure 4:**
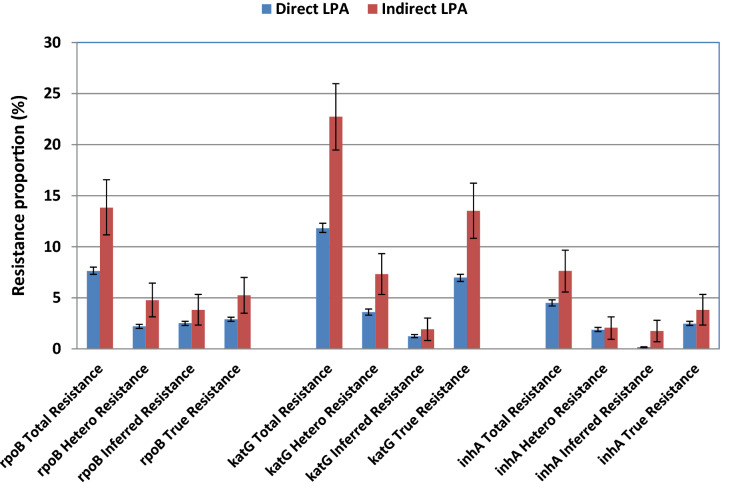
Comparison of Resistance detection by direct and indirect LPA.

From the matrix analysis of Rifampicin and Isoniazid resistance genes, it is observed that MUT3 (S450L) of *rpoB* and *katG*MUT1 (S315T1) pair contributed to 397 (36.6%; 95% CI: 33.8–39.5) MDR-TB cases (Table 6). Independently, *rpoB* MUT3 and katG MUT1 are associated with 52%, and 71.6% of MDR-TB cases. Of the 1084 MDR-TB cases, inferred resistance due to loss of wild type *rpoB*, *katG*, and *inhA* gene was found in 310 (28.6%), 98 (9%), and 17 (1.6%) cases, respectively. The Line probe assay was performed on direct positive sputum specimens in 19,614 (97%) of cases and from the culture in 604 (3%) cases. The relative proportions of resistant cases were compared across direct and indirect line probe assays (Fig. 4). The Indirect line probe assay nearly doubled the detection of drug resistance.

**Table 6:** Matrix analysis of pairing of rifampicin (*rpoB*) and isoniazid (*katG* and*inhA*) mutations in the emergence of MDR-TB.

Variables	katG WT	katG MUT1	katG MUT2	inhA WT1	inhA WT2	inhA MUT1	inhA MUT2	inhA MUT3A	inhA MUT3B	inhA WT 1&2	katG MUT1&inhA MUT1	katG MUT1&inhA MUT3A	katG MUT1 &inhA MUT3B	Total
WT1	3	0	1	0	0	0	0	0	0	0	0	0	0	4
WT2	2	29	0	2	0	6	0	0	0	0	0	0	0	39
WT2&3	1	20	1	1	0	4	0	0	0	1	0	0	0	28
WT3	2	8	0	0	0	0	0	0	0	0	0	0	0	10
WT4	0	1	0	0	0	0	0	0	0	0	0	0	0	1
WT3&4	9	47	1	0	0	7	0	0	1	0	0	0	1	66
WT5	0	1	0	0	0	0	0	0	0	0	0	0	0	1
WT4&5	0	5	0	0	0	2	0	0	0	0	0	0	0	7
WT6	0	0	0	0	0	0	0	0	0	0	0	0	0	0
WT5&6	2	10	0	1	0	3	0	0	0	0	0	0	0	16
WT7	9	44	1	0	0	5	0	1	0	5	0	0	0	65
WT8	6	54	0	1	0	9	0	0	0	1	1	0	1	73
MUT1	5	32	1	0	0	4	0	0	1	0	1	0	0	44
MUT2A	13	64	2	0	0	6	0	1	1	0	1	0	0	88
MUT2B	3	53	0	0	0	5	0	0	0	0	1	0	0	62
MUT3	39	397	2	4	0	97	0	2	4	1	15	1	2	564
MUT1&3	3	5	0	0	0	0	0	1	0	0	0	0	0	9
MUT2A&2B	1	6	0	0	0	0	0	0	0	0	0	0	0	7
Total	98	776	9	9	0	148	0	0	0	8	0	0	4	1084

## Discussion

India has the highest global burden of MTB and MDR-TB. Nearly half of the world's MDR-TB patients are from three countries, namely, India (27%), China (14%), and Russia (9%)^9^. Indian survey of TB drug resistance in 2016 reports a lower incidence of MDR in treated (11.6% vs. 18%) and new cases (2.84% vs. 3.4%) in comparison with the global WHO 2019 report^2^. This study observed a gradual decline in MDR-TB from 5.06% in 2015 to 1.34% in 2018, although this data pertains to presumptive MDR-TB cases. Mono-resistance to Rifampicin and Isoniazid also shows a decline from 2015 onwards, pointing to the effectiveness of the Revised National TB Control Program (RNTCP) in the state. About 60 to 70% of our rifampicin-resistant TB is multidrug-resistant, in close concordance with the global value of 78%^10,11^. The MDR-TB prevalence for untreated cases in our study is 6%, which is higher than the global (3.4%) and the national average (2.84%). The higher prevalence of MDR-TB may be explained by the patient selection bias involved, as only samples from suspected MDR-TB cases were sent to the reference laboratory. In this study, the majority of the suspected (52.5%) and lab-confirmed (56%) MDR-TB patients were from the age group between 15 and 45 years in concordance with the national data. The high frequencies of MDR-TB among young age groups may indicate the possibility of propagation of MDR-TB in the community because of the higher mobility of youth^12,13^.

Traditionally, the etiopathogenesis of MDR-TB is attributed to poor compliance and programmatic failure. We do not find any significant difference in the MDR-TB prevalence between previously treated and new cases. Although isoniazid mono-resistance had a small positive association with previous treatment, rifampicin mono-resistance was not associated with earlier treatment. Our results support the observation by Dheda et al. claiming that factors other than poor compliance and program failure are strongly implicated in the prevalence of MDR-TB, and they need to be identified^14^. There is an inherent male bias (1.9:1) in the incidence of tuberculosis, which may be attributed to a biological difference in response to mycobacterium. Post-pubertal females tend to mount a more robust  immune response leading to the tuberculoid form of cured or contained disease. This response may likely play a role in the pathogenesis of multidrug-resistance / rifampicin-resistance^15,16^. In our study, the proportion of female presenting with MDR-TB and non-MDR-TB is 19.3% (95% CI: 16.3–21.6) and 14.7% (95% CI: 14.2–15.2) respectively. A Chinese study by Liu et al. also attributes the positive association of MDR-TB with the female gender^17^. We also infer that old age and HIV positive individuals are less susceptible to MDR-TB from the multivariate logistic regression analysis. The waning immunity with age and compromised immune response in HIV may be attributed to the lower prevalence of MDR-TB in these groups. Although many studies have suggested the role of HIV in augmenting the prevalence of MDR-TB, our study using a reasonably large sample size did not find evidence for this observation^18–22^^.^ This may be attributed to behavioural differences rather than biological differences in studies conducted in a small population. It may also be attributed to regions with a higher prevalence of both HIV and MDR-TB. Our observation that HIV is not a MDR-TB risk factor is in concordance with the study by Baya et al. from Mali in 2019^23^. Our study also revealed a significant positive association between MDR-TB and treatment failure concordance with several other similar reports.

TB drug resistance may be sub-classified as true resistance, heteroresistance and Inferred resistance. In true resistance, only the mutant strain is present. Detection of both the mutant and the wild type strains is known as heteroresistance, while the absence of both mutant and wild type strain is considered as Inferred resistance. Heteroresistance is considered to be the early stage in the development of drug-resistant TB. Heteroresistance to *rpoB*, *katG*, and *inhA* by line probe assay in our study was 29.3%, 22.2%, and 40.7%, respectively. Heteroresistance, reflective of the slow evolution of bacteria from a sensitive to resistant profile, is a well-documented phenomenon in *M. tuberculosis*^24^. However, to our knowledge, only a few studies using a small number of cases have analysed this property. The clinical implications of heteroresistance are not fully ascertained. The sensitivity of GenoType MTBDRplus for detection of heteroresistance is reported to be about 5% from a mixed liquid culture^25^. Countries of the former Soviet Union have a higher proportion of MDR-TB (> 50%), as documented by WHO. A small study from Uzbekistan conducted in 2009 reports a 20% heteroresistance to rifampicin and/or isoniazid by direct LPA method. Another study from Mumbai, India (2012) based on Indirect LPA done on solid culture medium reports a rifampicin, isoniazid, and *inhA* heteroresistance of 34%, 39%, and 74%, respectively^26,27^. Although Rinder et al. claims that heteroresistance may be obscured by culture, we find that liquid culture enhances heteroresistance detection using GenoType MTBDRplus. Besides, the detection of total resistance and inferred resistance also were doubled by Indirect LPA assay in comparison with direct LPA on sputum specimens^28^. The rate of 72.5% of *katG* S315T mutation in non-MDR INH resistant isolates is an agreement to the finding by Manson et al., who reported a rate of 79%, and it is the harbinger mutations that often precede MDR^29^. In matrix analysis, 36.6% of strains carrying S315T1 mutations are associated with S450L mutations leading to MDR. We also observe that the single mutation in *katG*, S315T, accounted for the majority of isoniazid-resistance.

One of the limitations of our study is a bias associated with the selective analysis of presumptive MDR-TB. Therefore the prevalence data from this study may exaggerate the proportion of MDR-TB in the community. Our multivariate analysis did not include other behavioural factors that may be associated with MDR-TB.GenoType MTBDRplus assay is not sensitive to detect less than 5% heteroresistance in the population.

## Conclusion

This study shows a temporal decline in the MDR-TB prevalence from 2015 to 2018 in South Indian states of Tamil Nadu and Puducherry. Besides, the factors positively or negatively associated with drug resistance were arrived by analyzing a large number of samples. They hence may represent a less biased estimate of the actual underlying elements related to drug resistance. Our data do not support traditional views on treatment compliance and HIV in the etiopathogenesis of MDR-TB. A positive association of MDR-TB with female gender and negative association with HIV seropositivity and old age suggests mechanisms by which the immune system and sex hormones may be involved in the etiopathogenesis of MDR-TB. Studies on these aspects may pave the way for innovative approaches to target MDR-TB.
